# Risk Factors for Preoperative DVT in Elderly Patients with Hip Fractures

**DOI:** 10.3390/jcm15041481

**Published:** 2026-02-13

**Authors:** Noratep Kulachote, Pheeraphat Phoophiboon, Pongsthorn Chanplakorn, Norachart Sirisreetreerux, Nachapan Pengrung, Paphon Sa-ngasoongsong

**Affiliations:** 1 Department of Orthopedics, Faculty of Medicine Ramathibodi Hospital, Mahidol University, Bangkok 10400, Thailand; noratep.kul@mahidol.ac.th (N.K.); pheeraphat104@gmail.com (P.P.); pongsthornc@yahoo.com (P.C.); norachart.sis@mahidol.ac.th (N.S.); golfsome24@gmail.com (N.P.); 2 Chom Bueng Crown Prince Hospital, Ratchaburi 70150, Thailand

**Keywords:** hip fracture, elderly, preoperative, deep vein thrombosis, risk factors

## Abstract

**Background:** Deep vein thrombosis (DVT) is a common and potentially serious complication in elderly patients with hip fractures, as it may progress to pulmonary embolism. Despite advances in perioperative care, preoperative DVT remains an important clinical concern; therefore, in this study, we aimed to identify risk factors associated with preoperative DVT in elderly patients with hip fractures. **Methods:** A retrospective case–control study was conducted in patients aged > 60 years with hip fractures who had undergone preoperative Doppler ultrasonography between January 2015 and August 2024, while patients with prior or chronic DVT or incomplete medical records were excluded. Demographic, clinical, and laboratory data were collected, and uni- and multivariate logistic regression analyses were performed to identify independent predictors of preoperative DVT. **Results:** Of 669 eligible patients, 454 were included, and 23 (5.1%) were diagnosed with preoperative DVT. The mean age of the whole cohort was 79.5 years, and 70.7% were female. Univariate analysis revealed that thirteen predictors with *p* < 0.1 were associated with preoperative DVT, while through multivariate analysis, we identified four independent predictors: female sex (*p* = 0.02), active smoking (*p* = 0.01), Wells’ score ≥ 2 (*p* = 0.01), and elevated platelet-to-lymphocyte ratio (PLR) (*p* = 0.05). The model demonstrated good discriminative performance, with an AUC of 0.81. **Conclusions**: Preoperative DVT remains clinically significant in elderly patients with hip fractures. Female sex, active smoking, higher Wells’ score, and elevated PLR are independent predictors of this condition, so incorporating these factors into preoperative assessment may improve risk stratification and optimize Doppler ultrasonography use.

## 1. Introduction

Many countries are now transitioning into aging societies, leading to a continuous rise in the incidence of hip fractures, which constitute a significant public health concern, as they are associated with loss of independence, decreased quality of life, high disability rates, and increased mortality [1]. One of the most common and serious complications in elderly patients with hip fractures is deep vein thrombosis (DVT), which typically occurs in the deep veins of the lower extremities. The pathogenesis of DVT can be explained by Virchow’s triad, comprising vascular injury from the fracture, venous stasis due to reduced mobility and venous congestion, and a hypercoagulable state [2].

The reported incidence of DVT in patients with hip fractures ranges from 2.6% to 17.3% [3,4,5,6,7,8,9], and approximately 20% of patients with DVT may progress to pulmonary embolism (PE), which is associated with significantly increased mortality, particularly among elderly patients with multiple comorbidities [10]. Therefore, the prevention and preoperative diagnosis of DVT are crucial for reducing morbidity and mortality.

Through reviewing the existing literature, we highlighted several of their limitations [3,4,6,7,8,9,11,12,13,14,15,16,17,18]: most previous studies were conducted in small cohorts, focused on a limited number of risk factors, and lacked comprehensive analyses in elderly populations. In the present study, we therefore aimed to identify risk factors associated with preoperative DVT in elderly patients with hip fractures, with the goal of enhancing clinical understanding and informing optimal management strategies for this vulnerable population.

## 2. Materials and Methods

### 2.1. Study Design and Setting

This study was designed as a retrospective case–control study. Ethical approval was obtained from the Institutional Review Board of the Faculty of Medicine, Ramathibodi Hospital, Mahidol University (Approval No. MURA2024/664). Electronic medical records from January 2015 to August 2024 were retrospectively reviewed.

### 2.2. Participants and Study Group Definitions

The study population comprised patients aged > 60 years who were diagnosed with hip fractures, including femoral neck fracture, intertrochanteric fracture, and subtrochanteric fracture, identified using ICD-10 codes S72.0, S72.1, and S72.2, and who had undergone preoperative Doppler ultrasonography (DUS) of the lower extremities.

Cases were defined as patients diagnosed with preoperative lower-extremity DVT confirmed by DUS of both legs within 24 h after admission. Controls were defined as patients without evidence of DVT on preoperative DUS.

Patients were excluded if their medical records were incomplete or unavailable for review, if they had a history of DVT that was still under treatment or had been previously treated without documented radiologic resolution, or if they were diagnosed with chronic DVT.

### 2.3. Data Collection

Data were extracted retrospectively from electronic medical records and categorized into demographic, clinical, and laboratory variables. Demographic data included sex, age, height, weight, body mass index (BMI), American Society of Anesthesiologists (ASA) physical status classification, smoking history, and fracture location.

Clinical data and fracture-related variables comprised comorbid conditions (diabetes mellitus (DM), hypertension (HT), dyslipidemia (DLP), ischemic heart disease (IHD), stroke, chronic kidney disease (CKD), chronic obstructive pulmonary disease (COPD), malignancy, and prior deep vein thrombosis); preoperative DUS findings for DVT screening; the time interval from fracture occurrence to hospital admission; concomitant use of antiplatelet or anticoagulant medications; pre-injury mobility status (independent ambulation, assisted ambulation, or wheelchair use); duration of immobilization defined by the duration of total or near-total bed confinement; and thromboembolic risk assessment using Wells’ and Caprini scores, calculated at the time of admission.

Laboratory data at the time of admission included complete blood count parameters (hemoglobin/hematocrit, platelet count, mean platelet volume (MPV), and white blood cell count (WBC)); immune-inflammatory markers (systemic immune-inflammatory index (SII), neutrophil-to-lymphocyte ratio (NLR), monocyte-to-lymphocyte ratio (MLR), platelet-to-lymphocyte ratio (PLR), and platelet-to-albumin ratio (PAR)); serum electrolytes (sodium (Na) and potassium (K)); renal function tests (blood urea nitrogen (BUN), creatinine, and estimated glomerular infiltration rate (eGFR)); coagulation profile (prothrombin time (PT), activated partial thromboplastin time (aPTT), thrombin time (TT), and international normalized ratio (INR)); and D-dimer levels. The eGFR was recorded from automated reporting of our laboratory.

### 2.4. Statistical Analysis

Descriptive statistics were used to summarize baseline characteristics. Continuous variables are presented as the mean ± standard deviation (SD) or median with interquartile range (IQR), depending on data distribution, and were compared using the independent *t*-test or Mann–Whitney U test, as appropriate. Categorical variables are expressed as frequencies and percentages and were compared using the Chi-square or Fisher’s exact test.

Univariate analysis (UVA) with logistic regression was performed to identify potential risk factors associated with preoperative DVT, and variables with *p* < 0.10 in this analysis were entered into a multivariate analysis (MVA) with a stepwise approach to identify independent predictors. Results are reported as odds ratios with 95% confidence intervals. A two-sided *p*-value < 0.05 was considered statistically significant. Statistical analyses were performed using MedCalc software (version 23.2.7; MedCalc Software Ltd., Ostend, Belgium).

## 3. Results

### 3.1. Baseline Demographic and Clinical Characteristics

From January 2015 to August 2024, a total of 669 elderly patients with hip fractures were identified. All patients received routine preoperative DUS screening within 24 h after admission, and after excluding 12 patients with a prior diagnosis of deep vein thrombosis (DVT) and 203 patients with incomplete laboratory data (including duration of immobilization, coagulation profile, and d-dimer level), 454 patients were included in the final analysis. Overall, 23 of these patients (5.1%) were diagnosed with preoperative DVT. The study population had a mean age of 79.5 years and was predominantly female (70.7%). Baseline demographic characteristics, comorbidities, fracture locations, and prefracture ambulatory statuses were comparable between the DVT and non-DVT groups: the former group had a longer time from fracture to hospital admission, higher rates of immobilization exceeding 72 h, and higher Wells’ scores. Several laboratory parameters, including platelet count, MPV, SII, NLR, PLR, PAR, serum Na, creatinine, and eGFR, differed significantly between the two groups (Table 1).

### 3.2. Univariate Analysis (UVA) of Risk Factors

The UVA results are provided in Table 2. Univariate logistic regression analysis revealed 13 predictors with *p* < 0.1, including female gender, active smoking, concomitant use of antiplatelets and anticoagulants, immobilization for more than 72 h, Wells’ score ≥ 2, platelet count, MPV, SII, PLR, creatinine, albumin, PAR, and Na (Table 2).

### 3.3. Multivariate Analysis (MVA) of Risk Factors

From UVA, a total of 13 predictors (female gender, active smoking, antiplatelet and anticoagulant use, immobilization > 72 h, Wells’ score ≥ 2, platelet count, MPV, SII, PLR, creatinine, albumin, PAR, and Na) were analyzed using multivariate logistic regression analysis with stepwise selection. The results of the MVA are summarized in Table 3. Four independent risk factors were identified as being significantly associated with the occurrence of preoperative DVT in elderly patients with hip fractures, including female gender (OR = 5.10, 95% CI: 1.31–19.87, *p* = 0.02), history of active smoking (OR = 6.57, 95% CI: 1.48–29.06, *p* = 0.01), Wells’ score ≥ 2 (OR = 2.66, 95% CI: 1.29–5.47, *p* = 0.01), and elevated PLR (OR = 1.001, 95% CI: 1.000–1.002, *p* = 0.05). The multivariate logistic regression model demonstrated good discriminative ability, with an AUC of 0.81 (95% CI, 0.77–0.85).

## 4. Discussion

DVT remains one of the most common and serious complications following hip fracture in elderly patients. The authors of previous studies have reported a wide range of preoperative DVT incidence rates, varying from 2.6% to 17.3%, reflecting differences in population characteristics, diagnostic methods, and perioperative management strategies [3,4,5,6,7,8,9]. Our findings demonstrated that female patients exhibited a higher incidence of preoperative DVT compared with male patients, a result consistent with previous studies by Zhang et al. (2023) [8] and Zhuang et al. (2024) [9], and with the previous meta-analysis by Wang et al. [15]. The correlation between female gender and preoperative DVT might be explained by genetic differences and hormonal changes following and complications associated with menopause [15]. Hormonal changes after menopause, particularly the decline in estrogen, may contribute to endothelial dysfunction and prothrombotic tendencies. In addition, biological differences in platelet function may also play a role. Ranucci et al. (2019) reported that women exhibit higher platelet activity compared with men, suggesting a gender-related difference in platelet activation [19], which could explain the increased susceptibility of female patients to preoperative DVT following hip fracture.

Our results demonstrated that patients with a history of active smoking had a 6.57-fold higher risk of developing preoperative DVT compared with non-smokers, a finding consistent with those of previous studies by Niu et al. (2021) [6] and Zhang et al. (2014) [20], which also identified smoking as an independent risk factor for DVT. The underlying mechanisms by which smoking increases thrombotic risk are multifactorial. Smoking has been reported to elevate circulating coagulation factors, particularly plasma fibrinogen, thereby activating the intrinsic coagulation pathway. It also contributes to impaired fibrinolysis, endothelial injury, inflammatory activation, and increased blood viscosity, all of which promote a prothrombotic state [20,21]. Although smoking cessation can lead to a rapid decline in fibrinogen concentrations—reaching levels similar to those of non-smokers and providing long-term vascular benefits [22,23]—its effect in preventing preoperative DVT among hip fracture patients is likely limited, as coagulable and inflammatory responses after trauma remain markedly elevated during the 3-to-7-day preoperative period [24,25].

Wells’ score is a widely used clinical tool for estimating DVT probability and guiding subsequent diagnostic testing. A score of 2 or higher indicates that DVT is likely, prompting further evaluation with diagnostic ultrasound. Luksameearunothai et al. (2017) reported that a Wells’ score of ≥2 had a high specificity (80.5%) for predicting preoperative DVT among patients with hip fractures [5]. Similarly, in our study, we demonstrated that a Wells’ score of ≥2 was a strong independent predictor of preoperative DVT (OR = 2.66). These findings suggest that, in patients with hip fractures who have a Wells’ score of 0–1, the routine use of preoperative Doppler ultrasonography may not be necessary, as the negative predictive value is high (89%); therefore, limiting Doppler screening to patients with higher Wells’ scores could help to reduce unnecessary testing and minimize surgical delays in low-risk individuals.

The formation of DVT is closely associated with immune-inflammatory activation, involving cytokine cascades, endothelial dysfunction, and platelet hyperreactivity [26]. Peripheral blood-derived indices such as the SII, NLR, MLR, and PLR have emerged as accessible markers reflecting systemic inflammation and thrombotic tendency. Among these, PLR represents the interplay between thrombosis and inflammation, integrating platelet activation and lymphocyte suppression into a single parameter [17]. In trauma patients, thrombogenic potential is often enhanced due to elevated platelet counts and reduced lymphocyte levels secondary to systemic stress. In our study, we found that a high PLR was independently associated with an increased risk of preoperative DVT, corroborating the findings of previous investigations. Niu et al. (2022) found that elevated PLR and NLR predicted acute DVT after femoral neck fracture [27], reporting that a PLR value of 179–238 was associated with a 1.86-fold increased risk of DVT. However, PLR should be interpreted cautiously, as it is a nonspecific marker that can be influenced by various conditions such as infection, hematologic disorders, corticosteroid use, or malignancy. Despite this limitation, PLR remains a simple, inexpensive, and readily available biomarker that may help identify patients at increased risk of DVT in the preoperative period.

This study has several limitations: First, its retrospective design inherently limits our control over data completeness and potential confounding variables. Important factors, such as the precise timing and regimen of thromboprophylaxis, as well as concurrent inflammatory or infectious conditions, were not consistently documented and therefore could not be comprehensively analyzed. In addition, a considerable number of patients (*n* = 203) were excluded primarily due to incomplete or missing medical records, which may have introduced selection bias and model overfitting. Therefore, prospective studies employing standardized data collection and uniform clinical protocols are warranted to confirm and extend these findings. Second, although the overall cohort size was relatively large, the number of preoperative DVT cases was small. This may be attributed to improved accessibility and timely care for hip fracture patients, increased awareness of DVT complications among the clinical team, and implementation of effective prophylactic protocols. Consequently, the overall DVT prevalence has gradually declined, which may have limited the statistical power and stability of the multivariate estimates. Nevertheless, the model still demonstrated good discriminative ability, as reflected by the AUC value. Future investigations with larger sample sizes or multicenter collaborations are warranted to enhance the reliability and external validity of these findings. Finally, while hematologic indices such as the platelet-to-lymphocyte ratio (PLR) demonstrated independent predictive value, they remained nonspecific markers of inflammation. Further research should explore the dynamic biomarker changes and underlying mechanisms linking systemic inflammation to venous thrombosis in this patient population.

## 5. Conclusions

Preoperative DVT remains a significant complication in elderly patients with hip fractures. The results from this study demonstrated that female gender, active smoking, a Wells’ score ≥ 2, and an elevated PLR were independent predictors of preoperative DVT. Incorporating these factors into preoperative risk assessment may enhance diagnostic efficiency and reduce unnecessary Doppler screening in low-risk patients. Further multicenter studies are warranted to validate these findings and develop integrated predictive models combining clinical and inflammatory parameters to optimize early detection and prevention strategies.

## Figures and Tables

**Table 1:** Baseline demographic and clinical characteristics of elderly patients with hip fractures.

	DVT Group (*n* = 23)	Non-DVT Group (*n* = 431)	*p*-Value
Age, year ^a^	80.3 ± 10.2	79.4 ± 8.2	0.64
Male:female ^b^	3:20	130:301	0.10
Height, cm ^a^	156.4 ± 7.8	156.7 ± 8.4	0.89
Weight, kg ^a^	53.4 ± 10.3	54.5 ± 11.5	0.64
BMI, kg/m^2 a^	21.8 ± 3.8	22. 2± 4.3	0.66
ASA physical status (ASA ≤ 3: ASA ≥ 4) ^b^	16: 7	348: 83	0.30
Comorbid conditions ^c^			
DM	10 (43.5)	177 (41.1)	0.83
HT	16 (69.6)	339 (78.7)	0.30
DLP	12 (52.2)	220 (51.0)	1.00
IHD	3 (13.0)	92 (21.3)	0.44
Stroke	1 (4.3)	56 (13.0)	0.34
CKD	11 (47.8)	260 (60.3)	0.28
COPD	3 (13.0)	27 (6.3)	0.19
Cancer	4 (17.4)	43 (10.0)	0.28
Active smoking ^c^	3 (13.0)	19 (4.4)	0.09
Concomitant use of antiplatelets and anticoagulants ^c^	6 (26.1)	204 (47.3)	0.08
Prefracture ambulatory status ^c^			
Gait aid dependence	10 (43.5)	183 (42.5)	0.90
Fracture location ^c^			
Femoral neck	10 (43.5)	260 (60.3)	0.24
Intertrochanter	11 (47.8)	152 (35.3)	
Subtrochanter	2 (8.7)	19 (4.4)	
Time from fracture to admission, days ^d^	5 (0.25 to 19.25)	1 (0.00 to 6.00)	0.01 *
Immobilization > 72 h ^c^	16 (69.6)	157 (35.4)	0.003 *
Wells’ score ^d^	2 (1 to 2)	1 (1 to 2)	0.02 *
Caprini score ^d^	13 (12 to 15)	13 (11 to 14)	0.29
D-dimer ^d^	7745 (3809 to 18,482)	7644 (2753 to 24,131)	0.94
WBC ^d^	9.88 (7.67 to 13.05)	9.32 (7.43 to 11.78)	0.34
Neutrophil count (*×10^9^ cells/L) ^d^	8.04 (6.86 to 11.18)	7.54 (5.54 to 9.61)	0.25
Lymphocyte count (×10^9^ cells/L) ^d^	0.83 (0.58 to 1.42)	1.03 (0.74 to 1.47)	0.16
Monocyte count (×10^9^ cells/L) ^d^	0.55 (0.39 to 0.70)	0.57 (0.41 to 0.74)	0.49
Platelet count (×10^9^ cells/L)	271 (191 to 371)	213 (169 to 280)	0.04 *
MPV ^d^	8.4 (7.7 to 8.7)	8.8 (8.1 to 9.6)	0.03 *
SII (×10^9^ cells/L) ^d^	2664.0 (1460.7 to 5782.1)	1561.4 (946.5 to 2535.0)	0.01 *
NLR ^d^	12.4 (6.5 to 16.7)	7.4 (4.6 to 11.4)	0.02 *
MLR ^d^	0.7 (0.4 to 1.2)	0.5 (0.4 to 0.8)	0.28
PLR ^d^	299.0 (159.1 to 624.6)	203.1 (145.4 to 312.5)	0.04 *
Hemoglobin ^d^	10.9 (9.7 to 13.0)	11.3 (9.9 to 12.5)	0.58
Creatinine ^d^	0.78 (0.57 to 0.85)	0.86 (0.67 to 1.36)	0.03 *
eGFR ^d^	54.9 (36.4 to 71.9)	43.0 (28.4 to 60.9)	0.10
Albumin ^d^	31.2 (26.3 to 36.1)	33.6 (30.9 to 36.5)	0.08
PAR ^d^	8.25 (5.19 to 14.14)	6.26 (4.89 to 8.58)	0.04 *
PT ^d^	12.4 (11.9 to 13.1)	12.1 (11.5 to 13.0)	0.43
aPTT ^d^	25.6 (22.8 to 27.1)	25.7 (23.6 to 27.9)	0.41
INR ^d^	1.05 (0.99 to 1.11)	1.02 (0.96 to 1.10)	0.34
TT ^d^	15.1 (12.0 to 17.6)	17.1 (13.9 to 18.3)	0.09
Na ^d^	134 (132 to 138)	138 (135 to 141)	0.002 *
K ^d^	4.1 (3.9 to 4.6)	4.2 (3.9 to 4.5)	0.59

* Significant difference with *p* < 0.05, ^a^: value presented as mean ± standard deviation, ^b^: value presented as ratio, ^c^: value presented as number of cases, ^d^: median with interquartile range. DM: diabetic mellitus; HT: hypertension; DLP: dyslipidemia; IHD: ischemic heart disease; COPD: chronic obstructive pulmonary disease; WBC: white blood cell count; MPV: mean platelet volume; SII: systemic immune-inflammatory index; NLR: neutrophil-to-lymphocyte ratio; MLR: monocyte-to-lymphocyte ratio; PLR: platelet-to-lymphocyte ratio; PAR: platelet-to-albumin ratio; eGFR: estimated glomerular filtration rate; PT: prothrombin time; aPTT: activated partial thromboplastin; INR: international normalized ratio; TT: thrombin time; Na: sodium level; and K: potassium level.

**Table 2:** Univariate logistic regression analysis of risk factors for preoperative DVT in elderly patients with hip fractures.

Risk Factors	OR	95% CI	*p*-Value
Age	1.01	0.96 to 1.07	0.64
Female gender	2.88	0.84 to 9.86	0.09
Height	0.99	0.95 to 1.05	0.89
Weight	0.99	0.95 to 1.03	0.64
BMI	0.98	0.88 to 1.08	0.65
ASA grade ≥ 4	1.83	0.73 to 4.60	0.20
DM	1.10	0.47 to 2.57	0.82
HT	0.62	0.25 to 1.55	0.31
DLP	1.05	0.45 to 2.42	0.92
IHD	0.55	0.16 to 1.87	0.34
Stroke	0.29	0.04 to 2.21	0.23
CKD	0.60	0.26 to 1.40	0.24
COPD	2.24	0.63 to 8.03	0.21
Cancer	1.90	0.62 to 5.84	0.26
Active smoking	3.25	0.89 to 11.91	0.07
Concomitant use of antiplatelets and anticoagulants	0.39	0.15 to 1.02	0.05 *
Gait aid dependence	1.04	0.45 to 2.43	0.92
Intertrochanteric fracture	1.88	0.78 to 4.53	0.16
Time from fracture to admission	1.01	0.99 to 1.02	0.51
Immobilization > 72 h	3.99	1.61 to 9.91	0.003 *
Wells’ score ≥ 2	2.96	1.56 to 5.60	0.001 *
Caprini score	1.11	0.90 to 1.37	0.32
D-dimer	1	1.00 to 1.00	0.80
WBC	1.06	0.94 to 1.20	0.34
Neutrophil count	1.08	0.95 to 1.22	0.24
Lymphocyte count	0.66	0.29 to 1.50	0.32
Monocyte count	0.94	0.20 to 4.38	0.94
Platelet count	1.00	1.00 to 1.01	0.01 *
MPV	0.63	0.41 to 0.97	0.04 *
SII	1.00	1.00 to 1.0002	0.01 *
NLR	1.02	0.99 to 1.05	0.13
MLR	1.34	0.85 to 2.10	0.21
PLR	1.00	1.0005 to 1.0025	0.003 *
Hemoglobin	0.91	0.73 to 1.14	0.42
Creatinine	0.43	0.15 to 1.19	0.10
eGFR	1.01	0.99 to 1.03	0.17
Albumin	0.91	0.84 to 0.99	0.03 *
PAR	1.14	1.06 to 1.23	0.0003 *
PT	0.93	0.78 to 1.12	0.47
aPTT	0.96	0.86 to 1.07	0.44
INR	0.52	0.08 to 3.62	0.51
TT	0.93	0.83 to 1.04	0.21
Na	0.91	0.85 to 0.97	0.007 *
K	1.38	0.65 to 2.92	0.40

*: Significant difference with *p* < 0.05; DM: diabetic mellitus; HT: hypertension; DLP: dyslipidemia; IHD: ischemic heart disease; CKD: chronic kidney disease; COPD: chronic obstructive pulmonary disease; WBC: white blood cell count; MPV: mean platelet volume; SII: systemic immune-inflammatory index; NLR: neutrophil-to-lymphocyte ratio; MLR: monocyte-to-lymphocyte ratio; PLR: platelet-to-lymphocyte ratio; PAR: platelet-to-albumin ratio; eGFR: estimated glomerular filtration rate; PT: prothrombin time; aPTT: activated partial thromboplastin time; INR: international normalized ratio; TT: thrombin time; Na: sodium level; and K: potassium level.

**Table 3:** Multivariate logistic regression analysis identifying independent risk factors for preoperative DVT in elderly patients with hip fractures.

Risk Factors	OR	95% CI	*p*-Value
Female gender	5.1	1.31 to 19.87	0.02 *
Active smoking	6.57	1.48 to 29.60	0.01 *
Wells score ≥ 2	2.66	1.29 to 5.47	0.01 *
PLR	1.001	1.000 to 1.002	0.05 *
PAR	1.08	0.99 to 1.18	0.06
Na	0.92	0.85 to 1.00	0.06

* Significant difference with *p* < 0.05. PLR: platelet-to-lymphocyte ratio; PAR: platelet-to-albumin ratio; and Na: sodium level.

## Data Availability

The data presented in this study are available on request from the corresponding author due to participants’ privacy concerns.

## Abbreviations

The following abbreviations are used in this manuscript:

DVT	deep vein thrombosis
PE	pulmonary embolism
DUS	Doppler ultrasonography
DM	diabetes mellitus
HT	hypertension
DLP	dyslipidemia
IHD	ischemic heart disease
CKD	chronic kidney disease
ASA	American Society of Anesthesiologists
BMI	body mass index
WBC	white blood cell count
MPV	mean platelet volume
SII	systemic immune-inflammatory index
NLR	neutrophil-to-lymphocyte ratio
MLR	monocyte-to-lymphocyte ratio
PLR	platelet-to-lymphocyte ratio
PAR	platelet-to-albumin ratio
Na	sodium
K	potassium
eGFR	estimated glomerular filtration rate
PT	prothrombin time
aPTT	activated partial thromboplastin time
TT	thrombin time
INR	international normalized ratio
UVA	univariate analysis
MVA	multivariate analysis
